# *CD40* polymorphisms were associated with HCV infection susceptibility among Chinese population

**DOI:** 10.1186/s12879-019-4482-5

**Published:** 2019-10-15

**Authors:** Ting Tian, Peng Huang, Jingjing Wu, Chunhui Wang, Haozhi Fan, Yun Zhang, Rongbin Yu, Chao Wu, Xueshan Xia, Zuqiang Fu, Jun Li, Ming Yue

**Affiliations:** 1 Department of Epidemiology and Biostatistics, Key Laboratory of Infectious Diseases, School of Public Health, Nanjing Medical University, Nanjing, 211166 China; 2 Jiangsu Provincial Center for Disease Control and Prevention, Nanjing, 210009 China; 3 Chinese People’s Liberation Army Eastern Theater Disease Prevention and Control Center, Nanjing, 210002 China; 4 Nanjing Drum Tower Hospital, Clinical College of Nanjing Medical University, Nanjing, 210008 China; 5 Faculty of Life Science and Technology, Kunming University of Science and Technology, Kunming, 650500 China; 6 Department of Infectious Diseases, the First Affiliated Hospital of Nanjing Medical University, No. 300 Guangzhou Road, Nanjing, 210029 Jiangsu China

**Keywords:** Hepatitis C virus, CD 40, Genetic polymorphism, Susceptibility, Infection outcomes

## Abstract

**Background:**

CD40, encoded by *TNFRSF5*, participates in the survival of B cells, process of antigen presentation and generation of CD8+ T cell memory. It also has an important effect on HCV antiviral immune response. This study aims to investigate whether *TNFRSF5* gene polymorphisms are associated with HCV infection outcomes among Chinese population.

**Methods:**

Three single nucleotide polymorphism (SNPs) (rs1535045, rs1883832, rs4810485) on *TNFRSF5* were genotyped by TaqMan assay among Chinese population, including 1513 uninfected subjects, 496 spontaneous viral clearance subjects and 768 persistent HCV-infected subjects. Logistic analysis was used to compare these SNPs among different groups in this cross-sectional study. Functional annotations of the identified SNPs were further evaluated by bioinformatics analysis.

**Results:**

After adjusted by age, gender and routes of infection, the results of logistic analysis indicated that individuals carrying rs1535045 T allele had a higher risk to infect HCV compared with C allele (in recessive model, adjusted OR = 1.368, 95%CI = 1.070-1.749, *P* = 0.012). Subjects carried rs1535045 TT genotype were more likely to infect HCV than wild CC genotype (adjusted OR = 1.397, 95%CI = 1.078-1.809, *P* = 0.011). For rs1883832, T allele was significantly associated with an increased risk of HCV infection (in recessive model, adjusted OR = 1.337, 95%CI = 1.069-1.673, *P* = 0.011). Subjects with TT genotype had more possibility to infect HCV (adjusted OR = 1.351, 95%CI = 1.060-1.702, *P* = 0.015). In the stratified analysis, rs1535045 and rs1883832 were remained in various subgroups and the heterogeneity test showed no pronounced heterogeneity in any pairwise comparison (all *P* > 0.05). In addition, the results of the cumulative effects showed a tendency of that the more risk alleles (rs1535045 T and rs1883832 T) subjects carried, the more possibility of HCV infection exhibited (*P*<0.001). In haplotype analyses, compared with the CC haplotype, CT, TC and TT was correlated with an increased risk to infect HCV (*P* = 0.029, *P* = 0.047 and *P*<0.001, respectively).

**Conclusions:**

In conclusion, *CD40* polymorphisms were significantly associated with the susceptibility to HCV among Chinese populations.

## Background

It is estimated that more than 184 million people around the world are infected with hepatitis C virus (HCV). HCV infection has brought about large burden on many countries resulting from the highly correlated hepatic and extrahepatic disorders such as cirrhosis, hepatocellular carcinoma and hepatic failure [1]. Furthermore, current vaccines are not available for effective prevention of HCV infection [2].

As is known, host genetic factors play an important role in infection outcome of HCV and the response to antiviral therapies [3]. IL-28B rs12979860 and HLA class II rs4273729 are independently associated with spontaneous resolution of HCV infection [4]. Genome-wide association studies (GWASs) of patients with chronic hepatitis C have identified the association between IL28B variations, especially rs12979860 and rs8099917, and response to treatment with peg–interferon alpha and ribavirin [5, 6]. Therefore, it is still essential to figure out the atlas of disease related genetic factors.

CD40, also known as tumor necrosis factor receptor superfamily 5 (TNFRSF5), which is principally expressed on the B cells, participates in the survival of B cells, process of antigen presentation and generation of CD8+ T cell memory [7]. It also has an important effect on antiviral immune response. In HCV-associated chronic liver diseases, CD40 is highly expressed on liver tissues and has a key role in hepatocyte survival [8]. To be more specific, CD40 can inhibit HCV replication and mediate viral clearance, and disruptions by activated innate immune mechanism [9]. *CD40* gene polymorphisms have been found to be associated with many immune related disorders, for instance, Kawasaki Disease, systemic lupus erythematosus and lung cancer [10–12]. According to previous study, rs1535045 T allele was higher than C allele in Coronary Artery Disease (CAD) patients [12]. It also has been found that rs1883832 T allele was associated with higher risk of sepsis [13]. One meta-analysis showed a significant association between the *CD40* rs4810485 T allele and rheumatoid arthritis (RA) [14] However, so far, no relevant research has addressed the association between *CD40* genetic variants and HCV infection susceptibility or outcomes. Hence, this study aimed to explore the relationships between *CD40* genetic variants and HCV infection outcomes among a Chinese high-risk population, including rs1535045, rs1883832 and rs4810485.

## Methods

### Study subjects

This study incorporates three high risk populations up to 2777 subjects. We recruited 720 hemodialysis (HD) subjects from nine hospital hemodialysis centers from October 2008 to May 2015, 459 intravenous drug users recruited from Nanjing compulsory detoxification center from December 2008 to November 2012 and 1598 paid blood donors from six villages in Zhenjiang City from October 2008 to September 2016. Exclusion criteria are as follows: 1. concurrently infected with other virus (human immunodeficiency virus or hepatitis B virus); 2. suffered from any other liver diseases (e.g. alcoholic, autoimmune or metabolic liver diseases); 3. accepted any antiviral therapies during this whole study. All participants were categorized into three groups. Group A were health subjects who were tested both anti-HCV and HCV-RNA negative. Group B were the spontaneous clearance group who were tested anti-HCV positive and HCV-RNA negative. Group C were called persistent infection group whose anti-HCV and HCV RNA were both seropositive. It is worth noting that all serological results were verified by three independent experiments within six consecutive months.

Demographic data, dangerous behavior exposure and medical histories of HCV infection were collected through structured questionnaires designed by professionals. Only achieved the rigorous professional trainings could the investigators carry out the interview for each participant. Quality control was throughout the entire process of investigation in order to make sure that the collected data was true and reliable.

### Viral testing

After the interview, an approximately 10-mL morning fasting venous blood was collected from each participant. The serum and white blood cells were isolated at the speed of 4000 rpm for 10 min immediately and refrigerate at − 80 °C before using. Taking appropriate approaches for detection of anti-HCV antibodies, HCV-RNA and diverse HCV genotypes followed by the standard operating protocols. The reagents used for each step were the third-generation enzyme-linked immunosorbent assay (for anti-HCV antibody), Trizol LS Reagent (for HCV-RNA) and Murex HCV Serotyping 1–6 Assay ELISA kit (for HCV genotype).

### SNPs selection and genotyping

SNP searching strategies: 1) Candidate *TNFRSF5* tagSNPs were download on the 1000 Genomes Project SNP database (www.internationalgenome.org) and selected through Haploview 4.2 software, 2) Potential functional SNPs predicted by following databases (UCSC, HaploReg v4.1, GTEx Portal, SNP Function Prediction, and microRNA-related SNP), 3) Relevant SNPs discovered by others scholars associated with viral hepatitis or other liver and immune-related disorders, 4) According to the criteria, the minor allele frequency (MAF) of selected SNPs must be more than 5% among Chinese Han population. Finally, three SNPs (rs1535045, rs1883832 and rs4810485) were chosen into this study.

Genomic DNA of each subject was extracted from peripheral blood leukocytes by proteinase K and phenol-chloroform respectively, and further purified by ethanol precipitation. We used TaqMan allelic discrimination assay to genotype three SNPs on an ABI PRISM 7900HT Sequence Detection System (Applied Biosystems, Foster City, CA, USA). For quality control, two blank controls were set in each 384-well plate, and a 100% concordance was achieved in 10% random samples. The success rates of genotyping for selected SNPs were all above 95%. The samples failed for genotyping were excluded from the statistical analyses. The genotyping results were read by SDS 2.3 software (Applied Biosystems, Foster City, CA, USA). The primers and probes sequences for those SNPs were listed in Additional file 1: Table S1.

### Relevant public database

The genotype information of *TNFRSF5* in CHB population was downloaded from the 1000 Genomes Project Phase3 SNP database (www.internationalgenome.org). We have attempted to find expression quantitative trait loci (eQTL) evidence of positive loci based on the public GTEx Portal V6p database (http://www.gtexportal.org/). SNP functional annotations were performed on the HaploReg v4.1 database (http://archive.broadinstitute.org/mammals/haploreg/haploreg.php), SNP Function Prediction (https://snpinfo.niehs.nih.gov/snpinfo/snpfunc.html) and UCSC genome browser (https://genome.ucsc.edu/cgi-bin/hgGateway).

### Statistical analysis

For further susceptibility analysis, Group B and Group C were combined as HCV infected group, then compared with Group A which are health subjects. For HCV chronicity analysis, Group C was contrasted with Group B. Differences of the distribution in demographic and clinical characteristics were compared between subjects among three groups. Wherever appropriate, these differences were evaluated using Student’s *t* test, χ^2^-test, Kruskal–Wallis test or one-way analysis of variance. A goodness of fit χ^2^ test was used to test Hardy–Weinberg equilibrium (HWE) for each SNP among healthy subjects. Linkage disequilibrium (LD) parameters (r^2^ and D′) of selected SNPs in CHB population were estimated by Haploview software (version 4.2). The odds ratios and 95% confidence intervals were used to estimate the association between SNPs and the HCV infection outcomes by adjusting age, gender and routes of infection using both univariate and multivariate logistic regression models in this cross-sectional study. *P<*0.05 in a two-sided test was considered statistically significant. Bonferroni correction was used to conduct multiple comparisons among genotypes and the *P* value was adjusted to 0.0167 (0.05/3). All the analyses were carried out by Stata/SE (V.12.0 for Windows; StataCorp LP, College Station, TX, USA).

## Results

### General demographic information

Demographical and clinical characteristics of all subjects were listed in Table 1. The distribution of age and gender were balanced among Group A, Group B and Group C (all *P* > 0.05), while the ALT, AST and routes of infection were significantly different among three groups (all *P* < 0.001). HCV genotypes of partial HCV patients were statistically different between Group B and Group C (*P*<0.05). The three SNPs were all in accordance with Hardy-Weinberg equilibrium in the Group A (*P* = 0.553 for rs1535045, *P* = 0.130 for rs1883832 and *P* = 0.723 for rs4810485).

**Table 1:** Demographical and clinical characteristics among HCV control, spontaneous clearance and persistent infection populations

Variables	Group A (%)	Group B (%)	Group C (%)	*P*
	*n* = 1513	*n* = 496	*n* = 768	
Age (years, mean ± SD)	52.65 ± 13.30	51.12 ± 13.82	51.89 ± 12.37	0.076^a^
Gender				0.130^b^
Male	603 (39.9)	194 (39.1)	273 (35.5)	
Female	910 (60.1)	302 (60.9)	495 (65.5)	
ALT (U/L)				< 0.001^b^
< 40	1421 (94.9)	387 (78.2)	443 (57.8)	
≥40	77 (5.1)	108 (21.8)	323 (42.2)	
AST (U/L)				
< 40	1422 (95.1)	394 (80.9)	454 (60.0)	< 0.001^b^
≥40	74 (4.9)	93 (19.1)	303 (40.0)	
Route of infection				< 0.001^b^
HD	553 (36.5)	91 (18.3)	76 (9.9)	
IVDU	181 (12.0)	138 (27.8)	140 (18.2)	
PBD	779 (51.5)	267 (53.9)	552 (71.9)	
HCV genotypes				< 0.001^b^
1b	–	42 (26.3)	223 (46.1)	
Non-1b	–	118 (73.7)	261 (53.9)	

Abbreviations: *ALT* alanine transaminase, *AST* aspartate aminotransferase, *HCV* hepatitis C virus, *HD* hemodialysis, *IVDU* Intravenous drug user, *PBD* paid blood donors

Group A: controls; Group B: spontaneous clearance subjects; Group C: persistent infection patients

^a^ *P* value of one-way ANOVA among three groups

^b^ *P* value of χ^2^-test among three/two groups

### Association between polymorphisms in *CD40* gene and susceptibility to HCV infection

The distributions of rs1535045, rs1883832, and rs4810485 genotypes among Group A, Group B, and Group C were shown in Table 2. To explore the association between *CD40* polymorphisms and hepatitis C susceptibility, logistic regression methods were used by constructing three genetic models (additive, dominant and recessive). After adjusted by age, gender and routes of infection, the logistic regression analysis discovered that rs1535045 T allele was remarkably bound up with higher risk to infect HCV compared with C allele (in recessive model, adjusted OR = 1.368, 95%CI = 1.070-1.749, *P* = 0.012). Subjects carried rs1535045 TT genotype were more likely to infect HCV than wild CC genotype (adjusted OR = 1.397, 95%CI = 1.078-1.809, *P* = 0.011). For rs1883832, T allele was significantly associated with an increased risk of HCV infection (in recessive model, adjusted OR = 1.337, 95%CI = 1.069-1.673, *P* = 0.011). Subjects with TT genotype had higher possibility to infect HCV (adjusted OR = 1.351, 95%CI = 1.060-1.702, *P* = 0.015).

**Table 2:** Genotypes distributions of *TNFRSF5* SNPs among control, spontaneous clearance and persistent infection groups

SNPs (genotype)	Group A	Group B	Group C	Group (B + C)/Group A		Group C/Group B	
	n(%)	n(%)	n(%)	OR(95%CI)^a^	*P* ^a^	OR(95%CI)^b^	*P* ^b^
rs1535045							
CC	688 (46.5)	214 (43.9)	328 (43.6)	1.00	–	1.00	–
CT	648 (43.8)	211 (43.3)	322 (42.8)	1.043 (0.884-1.231)	0.618	0.979 (0.763-1.256)	0.867
TT	142 (9.7)	62 (12.8)	103 (13.6)	**1.397 (1.078-1.809)**	**0.011**	1.095 (0.760-1.576)	0.626
Dominant model				1.109 (0.948-1.297)	0.197	1.005 (0.796-1.271)	0.964
Additive model				1.135 (1.011-1.275)	0.032	1.027 (0.869-1.215)	0.752
Recessive model				**1.368 (1.070-1.749)**	**0.012**	1.106 (0.785-1.559)	0.564
rs1883832							
CC	567 (39.4)	178 (37.3)	275 (37.7)	1.00	–	1.00	–
CT	693 (48.2)	217 (45.5)	333 (45.6)	1.019 (0.858-1.210)	0.833	1.008 (0.778-1.306)	0.951
TT	178 (12.4)	82 (17.2)	122 (16.7)	**1.351 (1.060-1.722)**	**0.015**	0.980 (0.695-1.383)	0.909
Dominant model				1.090 (0.926-1.282)	0.300	1.001 (0.785-1.275)	0.997
Additive model				1.127 (1.004-1.263)	0.042	0.993 (0.841-1.173)	0.937
Recessive model				**1.337 (1.069-1.673)**	**0.011**	0.976 (0.713-1.335)	0.878
rs4810485							
GG	604 (40.6)	190 (39.7)	306 (41.0)	1.00	–	1.00	–
GT	693 (46.6)	211 (44.1)	328 (43.9)	0.970 (0.819-1.148)	0.721	0.981 (0.761-1.266)	0.885
TT	191 (12.8)	78 (16.2)	113 (15.1)	1.201 (0.944-1.527)	0.136	0.927 (0.655-1.312)	0.667
Dominant model				1.021 (0.871-1.196)	0.802	0.967 (0.762-1.227)	0.780
Additive model				1.063 (0.949-1.190)	0.290	0.967 (0.819-1.141)	0.688
Recessive model				1.220 (0.976-1.526)	0.081	0.936 (0.679-1.290)	0.685

Abbreviations: *CI* confidence interval, *HCV* hepatitis C virus, *OR* odds ratio, *SNP* single nucleotide polymorphism

Group A: controls; Group B: spontaneous clearance subjects; Group C: persistent infection patients. Group (B + C): Infected individuals

^a^The *P* value, OR and 95% CIs of Group (B + C) versus Group A were calculated on the basis of the logistic regression model, adjusted by gender, age and routes of infection

^b^The *P* value, OR and 95% CIs of Group C versus Group B were calculated on the basis of the logistic regression model, adjusted by gender, age and routes of infection

Bonferroni correction was applied and the *P* value was adjusted to 0.0167 (0.05/3)

Bold type indicates statistically significant results

### Association of *CD40* polymorphisms with the spontaneous clearance of HCV infection

When exploring the association of *TNRSF5* polymorphisms with the spontaneous clearance of HCV infection, we proceeded association study by comparing the distribution of genotypes of selected SNPs in the persistent infection group and spontaneous clearance group after the adjustment by gender, age and routes of infection. No significant association was discovered in any of the three SNPs (rs1535045, rs1883832 and rs4810485) with the virus spontaneous clearance both in the additive, dominant, recessive models (all *P*>0.05, Table 2).

### Stratified analysis

Considering that the genetic pattern of positive SNPs is recessive, we conducted the stratification analysis in recessive model (Table 3). We found that the rs1535045 were significant in female (adjusted OR = 1.639, 95%CI = 1.181-1.274, *P* = 0.003) and paid blood donors (adjusted OR = 1.417, 95%CI = 1.013-1.985, *P* = 0.042) subgroups after adjusted by age, gender and routes of infection. As for rs1883832, it remained meaningful in young (adjusted OR = 1.485, 95%CI = 1.020-2.162, *P* = 0.039), male (adjusted OR = 1.532, 95%CI = 1.077-2.180, *P* = 0.018), hemodialysis patients (adjusted OR = 2.042, 95%CI = 1.225-3.404, *P* = 0.006) and paid blood donors (adjusted OR = 1.836, 95%CI = 1.021-3.304, *P* = 0.043) subgroups. The heterogeneity test showed no pronounced heterogeneity in any pairwise comparison (*P* > 0.05).

**Table 3:** Stratified analysis of rs1535045 and rs1883832 among control, spontaneous clearance and persistent infection groups

SNPs	Allele	Subgroups	Group (B + C)/Group A		
			OR(95%CI)^a^	*P* ^a^	*P* ^b^
rs1535045	C/T	Age			
		<50	1.417 (0.973-2.064)	0.069	0.787
		≥50	1.323 (0.955-1.832)	0.092	
		Gender			
		male	1.240 (0.764-1.652)	0.551	0.158
		female	**1.639 (1.181-1.274)**	**0.003**	
		Routes of infection			
		HD	1.285 (0.684-2.413)	0.436	0.479
		Drug user	0.985 (0.607-1.598)	0.951	
		PBD	**1.418 (1.013-1.985)**	**0.042**	
rs1883832	C/T	Age			
		<50	**1.485 (1.020-2.162)**	**0.039**	0.572
		≥50	1.297 (0.979-1.719)	0.070	
		Gender			
		male	**1.532 (1.077-2.180)**	**0.018**	0.344
		female	1.228 (0.917-1.646)	0.168	
		Routes of infection			
		HD	**2.042 (1.225-3.404)**	**0.006**	0.066
		Drug user	**1.836 (1.021-3.304)**	**0.043**	
		PBD	1.111 (0.839 -1.471)	0.168	

Abbreviations: *CI* confidence interval, *HD* hemodialysis, *PBD* paid blood donors, *OR* odds ratio

Group A: controls; Group B: spontaneous clearance subjects; Group C: persistent infection patients. Group (B + C): HCV-infected individuals

^a^ The *P* value, OR and 95% CIs of group (B + C) versus Group A were calculated on the basis of the binary logistic regression model, adjusted by gender, age and routes of infection in recessive model (TT versus [TC + CC])

^b^ The *P* value for the heterogeneity test

Bold type indicates statistically significant results

### Cumulative effects of rs1535045 and rs1883832 on the susceptibility of HCV infection

The analysis of combined risk alleles (rs1535045 T allele and rs1883832 T allele) came to conclusions that subjects carried three risk alleles or four risk alleles were more likely to be infected when compared with subjects carried no risk alleles (adjusted OR = 1.947, 95%CI = 1.741-2.179, *P*<0.001, Table 4). Nevertheless, those carried one and two risk alleles showed no higher possibility to infect HCV than subjects who carried risk-free alleles. These two SNPs had trend influence on more possibility of being infected by HCV after the Cochran–Armitage trend test (*P*<0.001).

**Table 4:** Cumulative effects of combined risk alleles (rs1535045 T and rs1883832 T) on the susceptibility to HCV infection

Variables	Group A	Group B+ Group C	Group (B + C)/Group A	
	n	n	OR(95%CI)^a^	*P* ^a^
0	154	123	1	
1	619	444	0.965 (0.734-1.269)	0.799
2	615	550	1.179 (0.899-1.546)	0.233
3 4	29	64	**1.947 (1.741-2.179)**	**<0.001**
Trend				**<0.001** ^b^
0	154	123	1	
1-4	1362	1138	1.114 (0.862-1.440)	0.411

Abbreviations: *HCV* hepatitis C virus, *OR* odds ratio,*95% CI* 95% confidence interval

Group A: controls; Group B: spontaneous clearance subjects; Group C: persistent infection patients. Group (B + C): HCV-infected individuals

^a^Logistic regression model, adjusted for gender, age, and routes of infection

^b^Cochran–Armitage trend test

Bold type indicates statistically significant results

### Haplotype analysis

According to the information from Haploview software (version 4.2), the rs1535045 (C/T) and rs1883832 (C/T) were strongly linkage disequilibrium (D’ = 1.000, r^2^ = 0.283). As these two SNPs were both associated with increased risk of HCV infection, we evaluated the combined effects of these two SNPs on HCV susceptibility by haplotype analysis. As shown in Table 5, compared with most frequent CC haplotype, subjects carrying haplotype CT (adjusted OR = 1.161, 95%CI = 1.015-1.327, *P* = 0.029), TC (adjusted OR = 1.151, 95%CI = 1.002-1.323, *P* = 0.047) and TT (adjusted OR = 2.550, 95%CI = 1.665-3.905, *P*<0.001) were more likely to infect HCV.

**Table 5:** Haplotype analysis of rs1535045 and rs1883832 in the study population

Variables	Group A	Group B+ Group C	Group (B + C)/Group A	
	n	n	OR(95%CI)^a^	*P* ^a^
CC	985	728	1	
CT	1087	929	**1.161 (1.015-1.327)**	**0.029**
TC	927	789	**1.151 (1.002-1.323)**	**0.047**
TT	33	76	**2.550 (1.665-3.905)**	**<0.001**

Abbreviations: *HCV* hepatitis C virus, *OR* odds ratio, *95% CI* 95% confidence interval

Group A: controls; Group B: spontaneous clearance subjects; Group C: persistent infection patients. Group (B + C): HCV-infected individuals

^a^Logistic regression model, adjusted for gender, age and routes of infection

Bold type indicates statistically significant results

## Discussion

Up to now, the pathogenesis of HCV has not been illuminated clearly enough because a number of immune-related pathways and a series of genes involve in the process of HCV infection. Growing researches on HCV are paid attention to find more genetic variations in order to identify relevant biological markers of the disease. We explored the possible associations between three *CD40* SNPs (rs1535045, rs1883832 and rs4810485) and HCV infection outcomes. In this study, we found that genetic variants of *CD40* (rs1535045 T allele and rs1883832 T allele) were significantly associated with an increased risk of HCV infection among Chinese population at the fist time.

*TNFRSF5* encodes a costimulatory protein called CD40, which have been found on antigen presenting cells. The CD40 has been found to play an essential role in mediating a broad variety of immune and inflammatory responses including T cell-dependent immunoglobulin class switching, memory B cell development, and germinal center formation [7, 15]. Various cytokines can control the expression level of CD40, particularly the IFN-γ, IL-1β, and TNF-α enhancing the expression on monocytes [16], endothelial cells [17, 18], thymic epithelium [19] and in six HCC cell lines [20]. In HCV-associated chronic liver diseases, CD40 might have important effects on hepatocyte survival and death in HCV-associated chronic liver diseases [8]. Studies demonstrated that CD40 signal can activate NF-κB in a dose-dependent manner, then NF-κB inhibits Fas or Tumor Necrosis Factor Receptor (TNFR) mediated apoptosis by blocking the activation of caspase-8 [21, 22]. Specifically, CD40 is up-regulated on hepatocytes during infection with hepatitis C virus, can inhibit HCV replication and mediate viral clearance, and disruptions by activated innate immune mechanism [9].

In our study, we found rs1535045 (located in intron1 of *TNFRSF5*) and rs1883832 (located in 5’UTR of *TNFRSF5*) have an association with susceptibility to HCV infection. However, no significant association was found among the three candidate SNPs and the spontaneous clearance of HCV, which may be related to the smaller sample size included in the analysis. Therefore, this result should be further verified in large sample studies. These SNPs had been broadly researched in different genetic backgrounds. It turned out that rs1535045 had connection to many diseases, such as coronary artery calcification [23] and small cell lung cancer [11], and rs1883832 was related to systemic lupus erythematosus [24] and acute coronary syndrome [25].

**Fig. 1:**
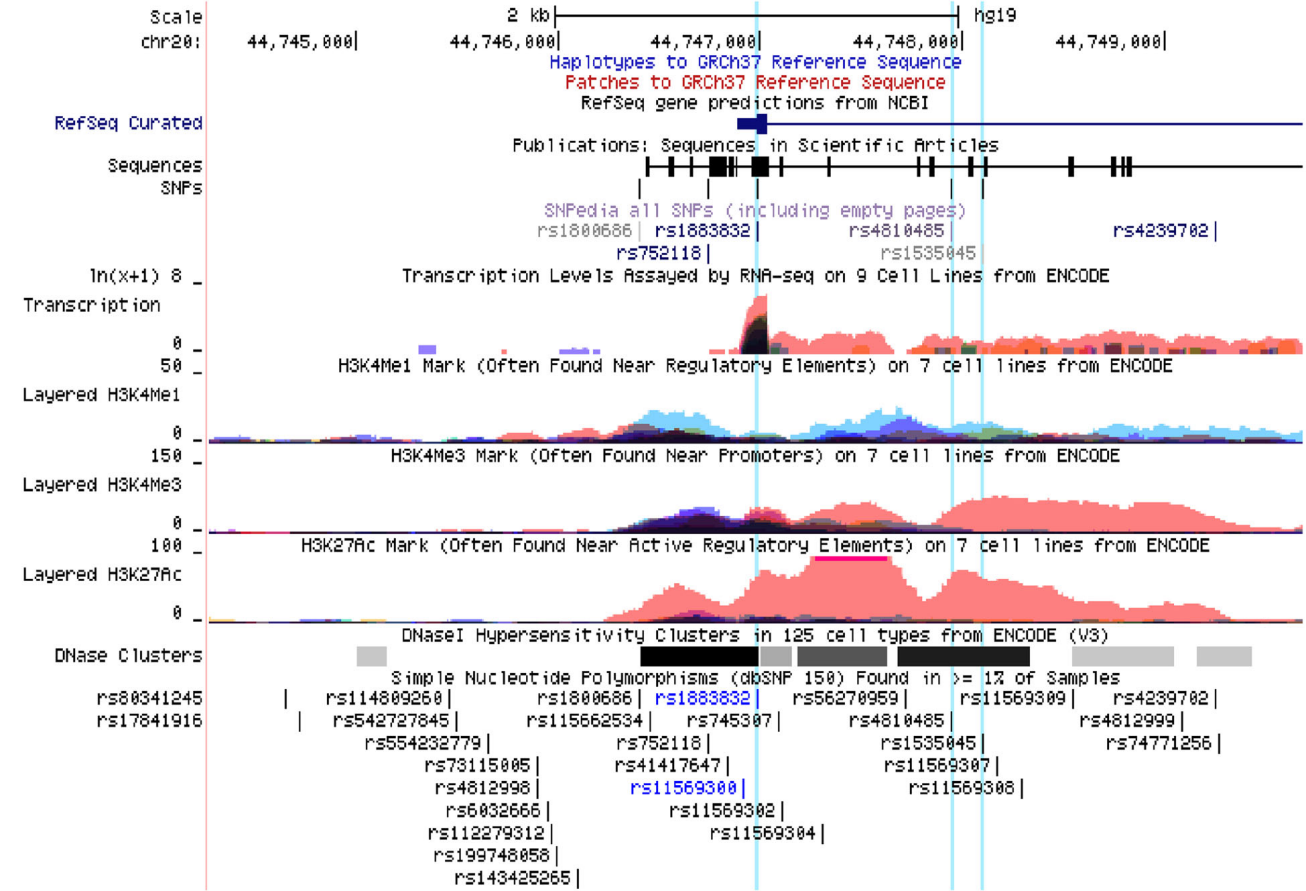
The functional annotations of rs1883832, rs4810485 and rs1535045 in UCSC genome browser were listed in this figure. SNPs marked with blue vertical lines (rs1883832, rs4810485, rs1535045 arranged from left to right) are located within the putative promoter transcription regulatory region characterized by Transcription Levels, histone protein modification (H3k4me1, H3k4me3 and H3k27AC) and DNase Hypersensitivity clusters

**Fig. 2:**
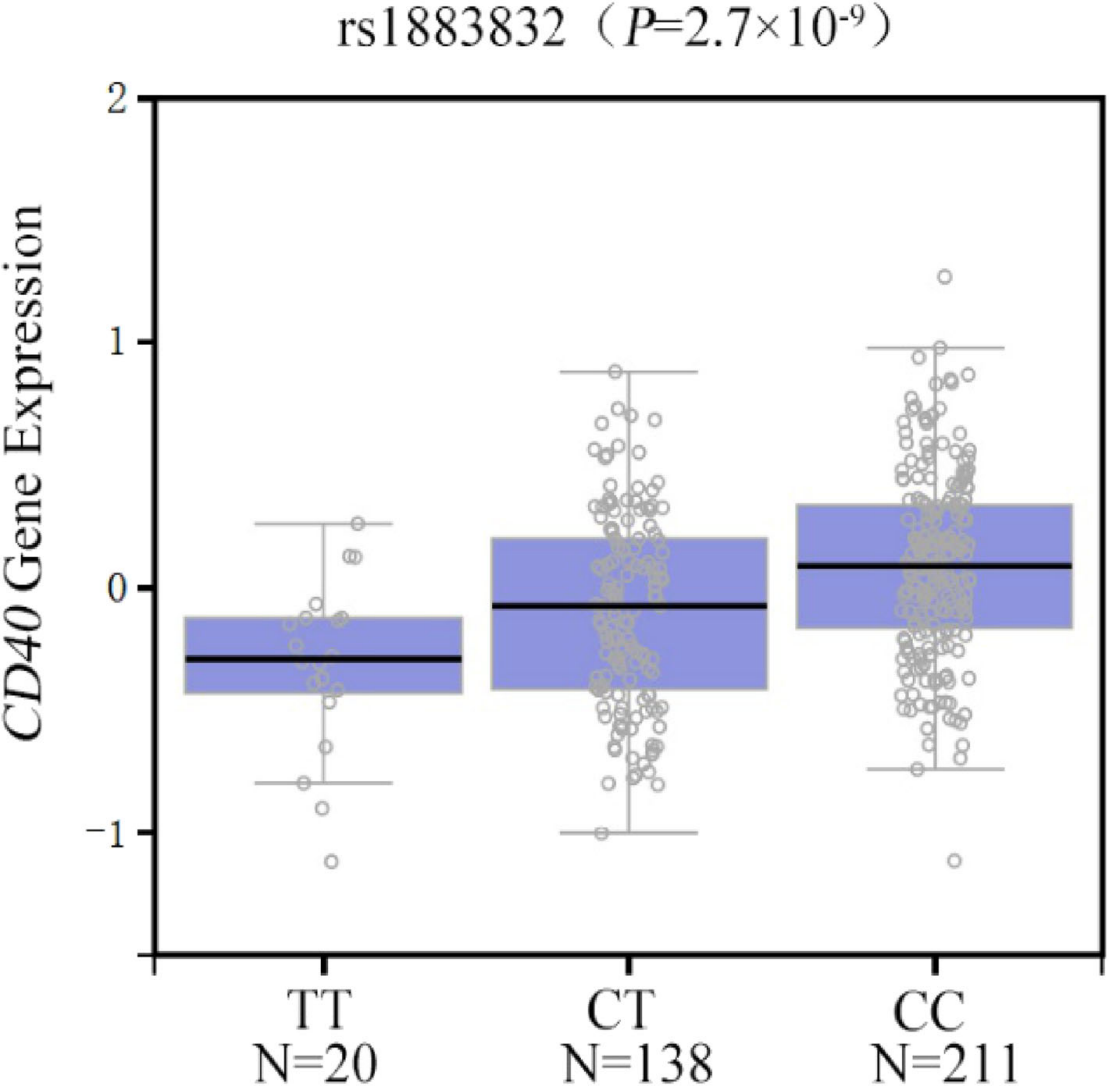
Expression quantitative trait loci (eQTL) analysis of rs1883832 with CD40 mRNA expression levels in the whole blood samples. The results showed that minor T allele of rs1883832 were pronouncedly associated with decreased expression levels of CD40. The *P* values were derived from linear regression model. This data was obtained from Genotype-Tissue Expression project (GTEx V6p) Portal

According to the bioinformatics information from UCSC, SNP rs1535045 was situated in highest peak of the H3k4me1, H3k4me3 and H3k27AC histone marker in 7 cell lines which were potential regulatory elements region (Fig. 1). We found rs1883832 was located at peak of transcription level assayed by RNA-seq on 9 cell lines from ENCODE and H3k27AC histone marker in 7 cell lines (Fig. 1). Recent studies have found that probable pathogenic SNPs are enriched in the enhancer region and regulatory elements region, particularly in the H3K27Ac region, indicating that these mutations may have possible functions on gene transcription and expression process and then effect the disease susceptibility and outcomes [26, 27]. Besides, we identified whether these SNP could cause any differential expression of mRNA in different tissues by eQTL analysis. It turned out that different genotypes of rs1883832 could cause differential mRNA level of CD40 in whole blood (*P* = 2.7 × 10^− 9^) (Fig. 2). Subjects carried the risk TT genotype had a lower CD40 mRNA levels, it was consistent with the results found by Rau SJ et al. that CD40 could inhibit HCV replication and mediate viral clearance, and disruptions [9].

In the stratified analysis, considering that the genetic pattern of positive SNPs is recessive, we conducted the analysis in recessive model. We found that rs1535045 and rs1883832 significantly increased the risk of HCV infection in some subgroups. Although different gender and route of infection can bring complex infection environment and immune response, rs1535045 T allele and rs1883832 T allele remained meaningful in various subgroups. In addition, the results of the cumulative effects and haplotype analyses showed a tendency of that the more risk alleles (rs1535045 T and rs1883832 T) subjects carried, the more possibility of HCV infection exhibited.

This research did have some limitations. First of all, the samples included in this study were poorly representative and lacked important information that might affect the outcome, such as HCV genotype and IL28B genotyping results. Second, this is a cross-sectional study and the association should be better verified in cohort study. Third, the evidence based on bioinformatics analysis was obtained on the computer simulation, further functional experiment should be carried out. In addition, this study only included high-risk populations of HCV infection, which may overestimate the association between candidate SNPs and the susceptibility of HCV infection. Therefore, our results must be verified in larger cohorts in the future.

## Conclusion

*CD40* polymorphisms was significantly associated with the susceptibility to HCV among Chinese populations. Further functional studies are needed to confirm the biological significance of these positive SNPs. This study provided the genetic evidence of association between CD40 polymorphisms and the outcome of HCV infection, and they may lead other scientists and clinician to identify new targets for therapy and personalized medical-based strategy by combined with other molecular genetics information.

## Supplementary information

**Additional file 1:** **Table S1.** Probes and primers of investigated *TNFRSF5* SNPs for TaqMan assay.

## Data Availability

The datasets used and/or analyzed during the current study are available from the corresponding author on reasonable request.

## Abbreviations

ALT: Alanine transaminase
AST: Aspartate aminotransferase
DAAs: Direct acting antivirals
eQTL: Expression Quantitative Trait Loci
HCV: Hepatitis C virus
LD: Linkage disequilibrium
MAF: Minor allele frequency
TNFRSF5: Tumor necrosis factor receptor superfamily 5
