# Supplementary material for: CD40 polymorphisms were associated with HCV infection susceptibility among Chinese population
Source: BMC Infect Dis. 2019 Oct 15;19:840. doi: 10.1186/s12879-019-4482-5 (PMC6792238; doi:10.1186/s12879-019-4482-5)
Supplement: Supplementary file 1 — Additional file 1: Table S1. Probes and primers of investigated TNFRSF5 SNPs for TaqMan assay. [file 12879_2019_4482_MOESM1_ESM.docx]

**CD40 polymorphisms affect the susceptibility to HCV infection among Chinese population**

**Table S1 Probes and primers of investigated *TNFRSF5* SNPs for TaqMan assay.**

| SNPs (Allele) | Gene | Region | MAF^a/b^ | *P^c^* | *P^d^* | TaqMan-MGB probe/primers sequences (5’-3’) |
| --- | --- | --- | --- | --- | --- | --- |
| rs1535045 | TNFRSF5(CD40) | intron | 0.315/0.400 | 0.553 | 0.378 | Forward primer: GCTCTTTCTCCACTCCTACCACAA |
| (C>T) |  |  |  |  |  | Reverse primer: TGGTCCCACAGCTTTCACTTT |
|  |  |  |  |  |  | Probe-T: FAM-TTTCCAGCTCCGCC-MGB |
|  |  |  |  |  |  | Probe-C: HEX-TCTTTCCAGCTCCACC-MGB |
| rs4810485 | TNFRSF5(CD40) | intron | 0.361/0.329 | 0.723 | 0.136 | Forward primer: TGGCCTCCCCCTACTTTAGAG |
| (G>T) |  |  |  |  |  | Reverse primer: CCTTGATACCATGGGTCATTCC |
|  |  |  |  |  |  | Probe-T: FAM-CTGTAGATTCCGGCCTG-MGB |
|  |  |  |  |  |  | Probe-G: HEX-CTGTAGATTCCTGCCTG-MGB |
| rs1883832 | TNFRSF5(CD40) | 5’UTR | 0.365/0.329 | 0.130 | 0.258 | Forward primer: AAAACAACTCACAGCGGTCAG |
| (C>T) |  |  |  |  |  | Reverse primer: GAAGACCCCGCCCCTTTC |
|  |  |  |  |  |  | Probe-T: FAM-ACGAACCATGGCGAG-MGB |
|  |  |  |  |  |  | Probe-C: HEX-ACGAACCATAGCGAG-MGB |

Abbreviations: SNPs, single nucleotide polymorphisms; MAF, minor allele frequency.

a minor allele frequencies in control group (Group A).

b minor allele frequencies from NCBI dbSNP database (available at https://www.ncbi.nlm.nih.gov/projects/SNP/snp).

c P value of Hardy-Weinberg equilibrium among control group (Group A).

d P value of Hardy-Weinberg equilibrium among spontaneous HCV clearance subjects (Group B).
